# Comparative Study Regarding the Adherence to the Mediterranean Diet Among Older Adults Living in Lebanon and Syria

**DOI:** 10.3389/fnut.2022.893963

**Published:** 2022-05-23

**Authors:** Joanne Karam, Carole Serhan, Eman Swaidan, Mireille Serhan

**Affiliations:** ^1^Department of Nutrition and Dietetics, Modern University for Business and Science, Beirut, Lebanon; ^2^Department of Business Management and Administration, University of Balamand, Beirut, Lebanon; ^3^Department of Nutritional Sciences, Faculty of Health Sciences, University of Balamand, Beirut, Lebanon

**Keywords:** MedDiet, dietary habits, older adults, Lebanon, Syria

## Abstract

The Mediterranean diet (MedDiet) has been associated with many health benefits. Poor adherence to MedDiet has been found among Lebanese adults, while in Syria, little is known about the adherence to MedDiet. A quantitative research approach was used, and data were collected through convenience sampling. The structure of the survey included the socio-economic and demographic data and the validated 14-point MedDiet assessment tool. The target population included 367 Lebanese and Syrian adults respectively residing in Lebanon and Syria. Descriptive statistics were used to explore the characteristics of the sample population. Adequate adherence MedDiet was determined if the Med-Diet score ≥ 9. Significant differences among the variables and the adherence to the MedDiet were examined using the chi-square test. Approximately 47.42% of participants reported adherence to MedDiet higher than 9, with a mean of 7.98. Lebanese participants, men, and those who are aged between 64 and 67, had higher adherence than Syrian participants, women, and other age groups, respectively. Lebanese participants (7.82 ± 2.32) had slightly higher adherence than Syrian participants (7.31 ± 2.04). Wine, *sofrito*, vegetables, and olive oil were mostly consumed by participants, with differences in consumption between the Lebanese and Syrian adults. The statistical analysis performed using the chi-square test showed no statistical difference (*P*>.05) between Lebanese and Syrian participants regarding their consumption of 160 red meat, butter/margarine, and sugary drinks. Future studies in the aged population are required to explore furthermore the adherence to MedDiet in Lebanon and Syria and its impact on health.

## Introduction

The Mediterranean Diet (MedDiet) represents a healthy dietary pattern in the context of healthy lifestyle habits. Epidemiological studies have shown that a higher degree of adherence to the MedDiet pattern is associated with reduced mortality concerning deaths due to cancer (1). Better control of diabetes through a protective role on glycemic control and a reduction in cardiovascular risk are also favored by the adoption of this dietary pattern (2). Moreover, MedDiet is significantly associated with less overweight and more weight loss (3).

In addition to its well-documented beneficial effects on health, MedDiet is considered a key component for healthy aging and might contribute to the onset of frailty at late stages of life (4). In older adults, the adherence to MedDiet moderates the association between multimorbidity and depressive symptoms (5). Greater adherence to a MedDiet was associated with better lower extremity physical performance in older adults with type 2 diabetes mellitus (T2DM) (6). Coelho-Júnior et al. (7) have investigated the association between adherence to MedDiet and physical performance and cognitive function in older adults (7). Results of the aforementioned study revealed that high adherence to MedDiet reduced the risk of global cognitive decline in non-demented older adults. Many observational studies led to the assumption that MedDiet could be a healthy eating pattern against cognitive and physical impairment in older adults (8–10).

Over the past few years, there has been a great effort to study the adherence to this Mediterranean-style diet, among the adult and older adult population in several Mediterranean and Gulf countries. In Spain, the influence of adherence to the MedDiet and its components on early vascular aging has been widely investigated (11). In Greece, adherence to the MedDiet is associated with higher levels of successful aging (12). In Italy, higher adherence scores were observed in females compared to males, and in elderly people compared to young people (13). Adherence to the MedDiet among the Cypriot adult population was associated with male sex, age >45 y, being married, being physically active, and being resident of rural regions (14). In Turkey, adherence to the MedDiet in hospitalized Turkish older adults and its association with hospital clinical outcomes was assessed (15). Moreover, in Gulf countries, the adherence to the MedDiet among adult participants from Saudi Arabia, Oman, and Kuwait was low (16).

In Lebanon, few studies were found to assess the compliance with the MedDiet (17–19). Poor adherence to MedDiet has been found among the Lebanese adult University students (20) and the adult population during the COVID-19 outbreak and the economic crisis (21). Other studies assessed the adherence to MedDiet in adolescents (22, 23) and among youth (24). However, in Syria, little is known about the adherence to MedDiet. (25) have evaluated the lifestyle factors, along with biomarkers related to cardiovascular risk factors in a group of university female students. The average adherence to the Mediterranean dietary pattern was low (25). Another study has assessed the Mediterranean adequacy index in young Syrians (26).

To our knowledge, no previous studies have focused on the compliance with the MedDiet among older adults in Lebanon and Syria and no previous study has compared the adherence to the MedDiet among the aforementioned countries. It is crucial to mention that adherence to the MedDiet might be useful for developing strategies for improving diet quality and setting governmental preventive health policies for the aged population. Therefore, the present study aimed to assess the adherence to the MedDiet among older adults from two Mediterranean countries (Lebanon and Syria) using a validated 14-item questionnaire.

## Materials and Methods

### Sampling Method

To overcome the practical difficulty of accessing participants from different geographical locations, a quantitative research approach was applied based on the distribution of an online survey throughout different social media platforms *via* networking. The target population of this research study included older Lebanese adults (45 and above) residing in Lebanon, and older Syrian adults (45 and above) residing in Syria. Lebanese and Syrian participants aged below 45 years old were not eligible to participate in the study, since they do not fit our age recommendations. A convenience sampling technique was used in this research.

### Population and Sample Size Calculation

The sample size was 367 participants from both countries (185 Lebanese vs. 183 Syrian). The sample size of 367 was considered enough, considering the target population of both the older Lebanese and Syrian adults, and through the determination with the sample size software, G^*^Power, version 3.1.3; margin of error of 5% confidence level of 95% (27). Data collection took place between July 2021 and December 2021.

### Ethical Considerations

This study received approval from the ethical committee of the Modern University of Business and Science (*approval reference MU-20210222-21, February 24th, 2021*). Older adult respondents provided written informed consent before filling out the questionnaire, and their confidentiality was protected. The collected data were solely used for scientific and research purposes.

### Study Tool

An online survey, created using Google Forms, was distributed. The structure of the survey included (1) the socio-economic and demographic data; (2) the validated Mediterranean Diet Assessment Tool. The validated Mediterranean diet (Med-diet) tool contained 14 items (1-point criteria for each item) to assess the participant's adherence to the diet, developed by the Prevención con Dieta Mediterránea (PREDIMED) consortium (28, 29). The score of adherence was calculated by summing the points of the 14 questions (“Yes” answer was one point, “No” was zero point; the results ranged between 0 and 14). The overall score of <9 points represented participants with low adherence, while the overall score of ≥ 9 points was used to identify participants with high adherence. This questionnaire has been adapted to and validated for the Spanish population (30) and then adapted in an English version to be implemented in other populations (31). The English version of the questionnaire was subsequently translated into Arabic. The latter is the native language of the participants. The questionnaire was piloted with 30 respondents (15 Lebanese vs. 15 Syrian) to determine the extent of their understanding of sentences and the time taken to answer questions. To determine the internal consistency of the survey questionnaire, a Cronbach's alpha coefficient reliability analysis was performed. This method shows an indication of the average correlation between all the items of the research questionnaire on the Likert scale, in this case. The Cronbach's alpha coefficient for the questionnaire was measured to be 0.960. The findings generated from the pilot study were not considered in the final data analysis.

### Data Analysis

Data were processed and analyzed by the SPSS statistical software, Windows Version 23.0 (SPSS, Inc., Chicago, IL, USA). Descriptive statistics including means of scores and standard deviations were used, to meet the study objectives. Frequencies were computed to examine the demographic and behavioral characteristics of the sample population. To determine their high adherence to Med-Diet, the Med-Diet score of ≥9 is used. A non-parametric test (chi-square) was employed to analyze the significant difference among the variables of gender, age, nationality, and adherence to the Mediterranean diet.

## Results

Mean adherence to Mediterranean Diet among the participants is shown in Table 1. Among the 367 participants, the mean MedDiet adherence score was 7.98 (± SD 2.11). Those who are aged between 64 and 67 had a higher mean (8.01 ± 2.27) than other age groups, followed by those aged 45 to 63 (7.48 ± 2.18). Men (8.53 ± 2.47) had a higher adherence compared to women (7.34 ± 2.29). Lebanese participants (7.82 ± 2.32) had slightly higher adherence than Syrian participants (7.31 ± 2.04).

**Table 1:** Mean adherence to mediterranean diet.

**Variables**		***N***	**%**	**Mean (SD)**
Total		367	100.0	7.98 (2.11)
Age (grouped data)	45-63	320	87.2	7.48 (2.18)
	64-67	19	5.2	8.01 (2.27)
	≥68	28	7.6	7.57 (2.35)
Gender	Men	129	35.1	8.53 (2.47)
	Women	238	64.9	7.34 (2.29)
Nationality	Lebanese	185	50.4	7.82 (2.32)
	Syrian	183	49.6	7.31 (2.04)

Table 2 shows the adequacy of adherence to the Mediterranean Diet according to different variables. About 47.42% of participants reported higher adherence to MedDiet than the adequate adherence score (≥ 9), while 52.58% of the participants had an adherence score lower than 9. Those who are aged between 45 and 63, as well as older than 68, showed similar adequacy of adherence (48.44% and 48.43%, respectively), higher than other age groups (64–67: 31.58%). Men (48.07%) and women (47.06%) reported similar adherence adequacy. Lebanese participants (49.73%) displayed higher adequacy of adherence than Syrian participants (45.06%). Specifically, Lebanese participants residing in Mount Lebanon (52.95%) and the North province (47.06) had lower adherence adequacy than other areas (64.29%). Results, however, were not significant (*p*> 0.05)

**Table 2:** Adequacy of adherence to MedDiet according to different variables.

**Variables**		**Score** ** <9**		**Score** **≥9**		p*	**Crude OR**
		***N***	**%**	***N***	**%**		**95% CI**
**Total**		193	52.58	174	47.42		
**Age**	45–63	165	51.56	155	48.44	0.25	0.60
	64–67	13	68.42	6	31.58		(0.56–0.64)
	≥68	15	53.57	13	48.43		
**Gender**	Men	67	51.93	62	48.07	0.28	0.68
	Women	126	52.94	112	47.06		(0.65–0.70)
**Nationality**	Lebanese	93	50.27	92	49.73	0.15	0.82
	Syrian	100	54.94	82	45.06		(0.72–0.91)
**Province in Lebanon**	Mount Lebanon	16	47.05	18	52.95	0.68	1.02
	North	72	52.94	65	47.06		(1)
	Others	5	35.71	9	64.29		

*OR, odds ratio; MedDiet, Mediterranean Diet; CI, confidence interval*.

*Evaluated by chi-square test.

Table 3 shows the stratification of adherence to Mediterranean Diet items. Most of the Lebanese and Syrian participants (78.74%) used olive oil as the main added fat, and 62.94% abided by the recommended quantity (four tablespoons or more). Vegetables (79.56%) were consumed by the participants according to MedDiet more than fruits (40.05%); 93.46% of the sample consumed wine, and 75.20% of the consumed legumes, according to the recommendations. Only 7.35% of the participants referred to an adequate intake of fish or shellfish. About 58.85% of the participants consumed commercial sweets <3 times/week, and 41.14% consumed the recommended portions of nuts per week. Additionally, chicken, turkey, or rabbit was preferred by more than half of the participants (58.58%) instead of beef, pork, hamburgers, or sausages. Consumption of *sofrito*, which is a sauce made with olive oil, onion, garlic, and tomato, was also very common among participants (83.92%).

**Table 3:** Stratification of adherence to MedDiet items.

	**All**				**Lebanese**				**Syrian**				
**Med-Diet Survey**	***N***	**%**	***N***	**%**	***N***	**%**	***N***	**%**	***N***	**%**	***N***	**%**	p*
	**Yes**		**No**		**Yes**		**No**		**Yes**		**No**		
Q1:Olive oil for cooking	289	78.74	78	21.26	157	84.86	28	15.14	132	72.52	50	27.48	0.04
Q2:Quantity of olive oil per day	231	62.94	136	37.06	111	60.00	74	40.00	120	65.93	62	34.07	0.02
Q3:Vegetables portion per day	292	79.56	75	20.44	142	76.75	43	23.25	150	82.41	32	17.59	0.03
Q4:Fruits portion per day	147	40.05	220	59.95	99	53.51	86	46.49	48	26.37	134	73.63	0.01
Q5:Red meat per day	266	72.47	101	27.53	120	64.86	65	35.14	146	80.21	36	19.79	0.18
Q6:Butter/ margarine portion per day	266	72.47	101	27.53	118	63.78	67	36.22	148	81.31	34	18.69	0.09
Q7:Sugary drinks portion per day	280	76.29	87	23.71	121	65.40	64	34.60	159	87.36	23	12.64	0.29
Q8:Wine portion per week	343	93.46	24	6.540	165	89.18	20	10.82	178	97.80	4	2,20	0.02
Q9:Legumes portion per week	276	75.20	91	24.80	148	80.00	37	20.00	128	70.32	54	29.68	0.03
Q10:Fish and fish products portion per Week	27	7.35	340	92.65	23	12.43	162	87.57	4	2.19	178	97.81	0.02
Q11:Processed deserts portion per week	216	58.85	151	41.15	106	57.29	79	42.71	110	60.43	72	39.57	0.03
Q12:Nuts portion per week	151	41.14	216	58.86	88	47.56	97	52.44	63	34.61	119	65.39	0.01
Q13:Consumption of chicken preferably over meat	215	58.58	152	41.42	107	57.83	78	42.17	108	59.34	74	40.66	0.05
Q14:Cooking vegetables, pasta, rice with olive oil, onion, garlic and tomato per week (*sofrito*)	308	83.92	59	16.08	160	86.48	25	13.52	148	81.31	34	18.69	0.01

*Lebanese vs. Syrian by chi-square test; MedDiet, Mediterranean diet.

Lebanese participants consumed more olive oil (84.86%), fruits (53.51%), legumes (80%), and fish products (12.43%) than Syrian participants according to the MedDiet recommendations (Table 3). Lebanese participants also showed more tendency toward nuts (47.56%) and *sofrito* (86.48%) consumption. However, more Syrian participants consumed the recommended daily quantity of olive oil (65.93%), preferred chicken over meat (59.34%), and consumed more vegetables (82.41%), wine (97.80%), and processed desserts (60.43%) than Lebanese participants. The statistical analysis performed using the chi-square test showed no statistical difference (*P*>.05) between Lebanese and Syrian participants regarding their consumption of red meat, butter/margarine, and sugary drinks (Q5, Q6, and Q7).

## Discussion

The mean adherence to the MedDiet in our study (7.98) was similar to the adherence in Spain (8.6), among Lebanese adults (8) and university students (7.96), but higher than that in the UK (5.47) and Korea (6.2). Little is known about the adherence to MedDiet in Syria. Less than half of the participants (47.42%) had an adequate score of adherence to MedDiet (≥9). This can be explained by the economic crisis hitting Lebanon and Syria, which led to a major increase in prices of traditional and healthy foods, forcing citizens to shift toward less expensive products and generally higher in calories, thus, less adhering to the MedDiet (32).

Those who are aged 64 to 67 had a higher mean (8.01) than other age groups, similar to earlier studies, where being older was related to higher adherence; this result suggests that MedDiet is rooted in cultural heritage and food traditions, maintained by older generations (13, 32, 33). Younger generations are, on the other hand, shifting toward unhealthier food patterns due to the widespread of non-Mediterranean food groups and Western diets (32, 33). However, the population above 68 showed a decrease in adherence (7.57), which may be the result of the age-related chemosensory changes and health problems, as deterioration in taste and smell senses and salivary function was observed in 60–70 years adults, leading to problems in flavor perception and consumption of unhealthy food as the last form of pleasure (34).

Differences in adherence were also seen among genders, where men had higher adherence than women This result is complementary to other studies, where females showed a lower adherence than males, among under 30-year-old Lebanese adults (35) and young Syrian people (26). This difference can be explained by the focus of women on body image, which leads to reducing calorie intake and healthy eating (36). The economic crisis also forced women to decrease their consumption of expensive products for the sake of their families (37), leading to a greater adherence among men, as higher adherence to MedDiet was associated with higher dietary costs, compared to lower costs for unhealthy foods (38).

Lebanese participants had slightly higher adherence than Syrian participants opposite to results obtained in 2004–2011, where Syria displayed a higher adherence than Lebanon (39). The current result can, thus, be explained by the increased food insecurity in Syria, which reached 55% of the population after the protracted economic crisis and war, compared to 34% among the Lebanese population, in late 2020 (40, 41).

Most of the participants used olive oil as the main added fat, mainly among Lebanese participants This high consumption of olive oil in the two countries is based on its wide production, as it forms a dominant agro-industrial product (42). Although this consumption was much higher in Syria than in Lebanon back in 2016 (43), the Syrian war caused a sharp fall in the olive oil sector, affecting Syrian nutritive intake (44).

According to MedDiet, the participants consumed vegetables more than fruits The number of fruits consumed was dominant among the Lebanese participants, while more Syrian participants consumed vegetables. Fruits became unaffordable upon the reduction of agriculture due to the Syrian war (44). The coronavirus disease 2019 (COVID-19) pandemic has also affected the transportation and production of fruits and vegetables in Lebanon, changing the food patterns of consumers involuntarily (45).

Roughly 93.46% of the sample consumed wine, according to the MedDiet recommendations, Syrian participants might have also consumed alcohol as a coping strategy to the war situation, as psychological stress might cause increased consumption of wine, although more evidence is needed on the impact of the crisis on alcohol drinking. However, only 7.35% of the participants referred to an adequate intake of fish, which was higher in Lebanon. This result confirms the outcome of earlier studies showing a low consumption of fish products among the Lebanese adult population (35, 46). Around 41.14% of participants also consumed nuts, as consumers tended to reduce their consumption of fish and nuts due to their unaffordable prices (37).

On the other hand, 75.20% consumed legumes, mainly among Lebanese participants. As a result of the COVID-19 lockdowns in Lebanon, dietary diversity was declined, and cooking practices increasingly relied on grains, pasta, and legumes due to their low prices and high stocking (45). Also, Syrian adults traditionally consume pulses as their main source of energy, mostly among low-income populations (26).

About 58.85% consumed commercial sweets, and Syrian participants showed more inclination toward processed desserts consumption This increased consumption was another consequence of the COVID-19 pandemic, mainly among food insecure consumers who suffer from mental problems and anxiety (45).

Additionally, chicken was preferred by more than half of the participants over meat, primarily among the Syrian population This result is similar to other studies showing that Syrian populations are having a higher tendency toward protein intake, which is mainly chicken (26). Consumption of *sofrito* was also quite common among participants Cooking using *sofrito* is very usual among Mediterranean people as it is a typical Mediterranean sauce, strongly associated with a reduced risk of CVD (46).

Consequently, this study showed that although MedDiet is traditionally prevailing in Lebanon and Syria, some of its components might be affected by the economic crisis. The association between income and adherence has been recently studied among Lebanese adults during the COVID-19 outbreak and the current economic crisis; participants with average to high income had higher adherence mean compared to other participants with lower salaries (21).

However, more comprehensive research is needed to better investigate the food patterns and MedDiet adherence of adults, mainly among the Syrian population.

### Strengths and Limitations

Mediterranean Diet has been assessed in Lebanon among the Lebanese young population. No previous studies have focused on the compliance with the MedDiet among older adults in Lebanon and Syria and no previous study has compared the adherence to the MedDiet among the aforementioned countries. Findings from this study might be useful for developing strategies for improving diet quality and setting governmental preventive health policies in the aged population. Moreover, our study includes several limitations that need to be acknowledged. First, the MedDiet assessment tool was conducted using a 14-question questionnaire validated among the Spanish population not among Lebanese and Syrian adults; however, the questionnaire was adapted to an English version used in other samples of young adults. Second, data were self-reported through an online survey, which might affect the accuracy of the results. Finally, this is a cross-sectional study, and, therefore, we acknowledge that we are not able to draw causal conclusions but only observations.

## Conclusion

Around 47.42% of participants reported adherence to MedDiet higher than 9, with a mean of 7.98. Lebanese participants, men, and those who are aged between 64 and 67 had higher adherence than Syrian participants, women, and other age groups, respectively. Wine, *sofrito*, vegetables, and olive oil were mostly consumed by participants, with differences in consumption between the Lebanese and Syrian adults. However, the lack of studies in Syria emphasizes the need for further research on the dietary habits among its population. Awareness and policies must be also spread at national levels to improve the public adherence to MedDiet amid the crisis, thus, ensuring better health outcomes and economic sustainability.

## Data Availability Statement

The raw data supporting the conclusions of this article will be made available by the authors, without undue reservation.

## Ethics Statement

The studies involving human participants were reviewed and approved by Modern University of Business and Science Ethics Committee (approval reference MU-20210222-21, February 24th, 2021). The patients/participants provided their written informed consent to participate in this study.

## Author Contributions

MS and JK designed the study, wrote the protocol, conducted literature searches, and provided summaries of previous research studies. ES collected data. CS conducted the statistical analysis. All authors wrote the first draft of the manuscript, read, and agreed to the published version of the manuscript.

## Conflict of Interest

The authors declare that the research was conducted in the absence of any commercial or financial relationships that could be construed as a potential conflict of interest.

## Publisher's Note

All claims expressed in this article are solely those of the authors and do not necessarily represent those of their affiliated organizations, or those of the publisher, the editors and the reviewers. Any product that may be evaluated in this article, or claim that may be made by its manufacturer, is not guaranteed or endorsed by the publisher.
